# Gliotoxin Targets Nuclear NOTCH2 in Human Solid Tumor Derived Cell Lines *In Vitro* and Inhibits Melanoma Growth in Xenograft Mouse Model

**DOI:** 10.3389/fphar.2017.00319

**Published:** 2017-07-07

**Authors:** Rainer Hubmann, Wolfgang Sieghart, Susanne Schnabl, Mohammad Araghi, Martin Hilgarth, Marlies Reiter, Dita Demirtas, Peter Valent, Christoph Zielinski, Ulrich Jäger, Medhat Shehata

**Affiliations:** ^1^Department of Internal Medicine I, Division of Hematology and Hemostaseology, Medical University of ViennaVienna, Austria; ^2^Department of Internal Medicine III, Division of Gastroenterology and Hepatology, Medical University of ViennaVienna, Austria; ^3^Department of Medicine I, Ludwig Boltzmann Cluster Oncology, Medical University of ViennaVienna, Austria; ^4^Comprehensive Cancer Center Vienna, Drug and Target Screening Unit, Medical University of ViennaVienna, Austria

**Keywords:** NOTCH2, gliotoxin, γ-secretase inhibitors, melanoma, hepatocellular carcinoma, pancreas carcinoma

## Abstract

Deregulation of NOTCH2 signaling is implicated in a wide variety of human neoplasias. The current concept of targeting NOTCH is based on using gamma secretase inhibitors (GSI) to regulate the release of the active NOTCH intracellular domain. However, the clinical outcome of GSI remains unsatisfactory. Therefore we analyzed human solid tumor derived cell lines for their nuclear NOTCH activity and evaluated the therapeutic potential of the NOTCH2 transactivation inhibitor gliotoxin in comparison to the representative GSI DAPT. Electrophoretic mobility shift assays (EMSA) were used as a surrogate method for the detection of NOTCH/CSL transcription factor complexes. The effect of gliotoxin on cell viability and its clinical relevance was evaluated *in vitro* and in a melanoma xenograft mouse model. Cell lines derived from melanoma (518A2), hepatocellular carcinoma (SNU398, HCC-3, Hep3B), and pancreas carcinoma (PANC1) express high amounts of nuclear NOTCH2. Gliotoxin efficiently induced apoptosis in these cell lines whereas the GSI DAPT was ineffective. The specificity of gliotoxin was demonstrated in the well differentiated nuclear NOTCH negative cell line Huh7, which was resistant to gliotoxin treatment *in vitro*. In xenotransplanted 518A2 melanomas, a single day dosing schedule of gliotoxin was well tolerated without any study limiting side effects. Gliotoxin significantly reduced the tumor volume in early (83 mm^3^ vs. 115 mm^3^, *p* = 0.008) as well as in late stage (218 mm^3^ vs. 576 mm^3^, *p* = 0.005) tumor models. In conclusion, NOTCH2 appears to be a key target of gliotoxin in human neoplasias and gliotoxin deserves further evaluation as a potential therapeutic agent in cancer management.

## Introduction

The highly conserved NOTCH gene family (NOTCH1-4) encodes trans-membrane receptors that regulate embryonic development and adult tissue homeostasis by modulating binary cell fate decisions in response to external signals (Louvi and Artavanis-Tsakonas, 2012; Ntziachristos et al., 2014). After ligand binding, canonical NOTCH signaling is initiated by a series of proteolytic events involving γ-secretase leading to the release of the NOTCH intracellular domain (N**^IC^**). N^IC^ translocates to the nucleus where it acts as context dependent transcription factor on CSL (for CBF1/Suppressor of Hairless/LAG-1) responsive genes (Louvi and Artavanis-Tsakonas, 2012; Ntziachristos et al., 2014).

NOTCH receptors act as tumor initiating oncogenes by rendering transformed cells into a less differentiated, immortalized state (Espinoza and Miele, 2013; Andersson and Lendahl, 2014). Deregulation of NOTCH2 signaling is observed in an increasing number of human neoplasias including chronic lymphocytic leukemia (CLL) (Hubmann et al., 2002; Rosati et al., 2009), marginal zone lymphoma (MZL) (Kiel et al., 2012), pancreas carcinoma (pancreas-CA) (Mazur et al., 2010; Zhou et al., 2013; Liu et al., 2017), hepatocellular carcinoma (HCC) (Dill et al., 2013; Hayashi et al., 2015; Huntzicker et al., 2015; Zhu et al., 2015; Wu et al., 2016), bladder cancer (Hayashi et al., 2016), medulloblastoma (Fan et al., 2004), glioblastoma (Yu et al., 2015), and melanoma (Hoek et al., 2004; Kaushik et al., 2014). It has been recently shown, for instance, that the nuclear NOTCH2 activity is functionally linked with the self-renewing capacity (stemness) and severity of liver cancer cells making nuclear NOTCH2 an ideal candidate for therapeutic interventions (Zhu et al., 2015).

Truncated, ligand independent NOTCH2 proteins are not tethered to the plasma membrane and, thus, do not require γ-secretase for processing and function (Lauring and Overbaugh, 2000). As a consequence, the nuclear NOTCH2 activity might be resistant to γ-secretase inhibitors (GSI) (Das et al., 2004), a phenomenon that we have observed in the majority of CLL cases (Hubmann et al., 2010, 2013). In light of the observation that GSI are less effective in clinical studies (Andersson and Lendahl, 2014; Lee et al., 2015), we hypothesize that GSI resistance might be a widespread characteristic of NOTCH2 associated human malignancies. Therefore, we tested human cell lines derived from melanoma, HCC, and pancreas-CA for their nuclear NOTCH activity by EMSA and evaluated their sensitivity to the representative GSI DAPT and to the *Aspergillum* derived canonical NOTCH2/CSL transactivation inhibitor gliotoxin which efficiently induced apoptosis in CLL cells (Hubmann et al., 2013). The secondary metabolite gliotoxin was identified as major virulence factor in *Aspergillosis* patients with immunosuppressive functions and since the discovery of its structure in 1958 (Bell et al., 1958), it became a target for extensive investigations to explore its complex mechanism of action and its multiple downstream effector molecules and for potential drug development (Gardiner et al., 2005; Dolan et al., 2015; Scharf et al., 2016).

## Materials and Methods

### Chemical Reagents, Compounds, and Culture

DAPT and gliotoxin, were obtained from Merck Millipore (Darmstadt, Germany). The compounds were reconstituted in dimethyl sulfoxide (DMSO). Human cell lines derived from melanoma (518A2), HCC (HEP3B, SNU398, Huh7), pancreas-CA (PANC1), and breast-CA (HCC38, MDA-MB-468) were cultured in RPMI 1640 supplemented with 10% heat inactivated fetal calf serum (FCS), 2 mM Glutamine, 100 U/ml penicillin, and 100 mg/ml streptomycin (all reagents were obtained from Gibco, Life Technologies Inc., Paisley, United Kingdom). HEP3B (HB-8064), SNU398 (CRL-2233), PANC1 (CRL-1469), HCC38 (CRL-2314), and MDA-MB-468 (HTB-132) cell lines were obtained from the American Type Culture Collection (ATCC, Rockville, MA, United States). The cell line Huh7 (JCRBO 403) was obtained from the National Institute of Biomedical Innovation (Osaka, Japan). The melanoma cell line 518A2, characterized by the *BRAF* V600E mutation and a *CDKN2A* exon 2 deletion, was obtained from Leiden University. The generation of the HCC cell line HCC-3 was described previously (Winter et al., 2008). Cells were incubated with the indicated concentrations of inhibitors or with equal amounts of solvent.

### Flow Cytometry and Detection of Cell Viability

Flow cytometry was performed on a FACSCalibur using CellQuest Pro software version 5.2.1 (Becton Dickinson, San Jose, CA, United States). Annexin V, and propidium iodide staining was performed to estimate the percentages of cells undergoing apoptosis using a kit from Bender Med. Systems Inc. (Vienna, Austria). The percentage of apoptotic cells was calculated as sum of propidium iodide (PI)-/Annexin V (Ax)+ (early apoptosis) and PI+/Ax+ (late apoptosis/necrosis) cells.

### Preparation of Nuclear Extracts, EMSA, and Western Blotting

3 × 10^7^ cells were lysed in 1 ml hypotonic buffer (10 mM HEPES, pH 7.9; 1.5 mM MgCl_2_; 10 mM KCL) containing 0.15% NP-40 at 4°C for 10 min. The nuclear proteins were extracted from the nuclear fraction by suspending the nuclei in 100 μl extraction buffer (300 mM KCl; 1.5 mM MgCl_2_; 20 mM HEPES, pH 7.9; 0.2 mM EDTA; 25% Glycerin) at 4°C for 20 min with constant agitation. A CSL site spanning oligonucleotide (5′-CAGCCCT*GTGGGAA*CTTGCTG-3′) was annealed with the reverse complementary strand and served as probe. EMSA for the detection of NOTCH/CSL complexes were performed essentially as described (Hubmann et al., 2002).

The N1^IC^ (bTAN 20) and N2^IC^ (C651.6DbHN) antibodies used for supershift/interference and western blot assays were obtained from the Developmental Studies Hybridoma Bank (University of Iowa, Department of Biological Science, Iowa City, IA, United States). The NFκB p65 (RelA) and ACTB Antibodies were purchased from Santa Cruz Biotechnology (Santa Cruz, CA, United States). Western blotting was performed according to standard protocols.

### Tissue Array Immunohistochemistry

For NOTCH2 cellular localization, human HCCs tissue array slides were obtained from SuperBioChips Laboratories, Seoul, South Korea. Heat induced antigen retrieval was performed in 10 mM citrate buffer at pH 6. After incubation with the NOTCH2 primary antibody (C651.6DbHN), slides were incubated with biotinylated secondary antibody, followed by alkaline phosphatase-streptavidin and chromogen. The stained slides were evaluated by a pathologist to evaluate NOTCH2 localization.

### Reverse Transcription Polymerase Chain Reaction (RT-PCR) Analysis

Total RNA was extracted using the TRI Reagent^®^isolation system (Sigma–Aldrich, St Louis, MO, United States). Moloney murine leukemia virus (M-MLV) reverse transcriptase and GoTaqPCR kits (Promega) were used for semiquantitative RT-PCR using primer sets as follows: *HEY1*, forward 5′-ATACGCCTGCATTTACCAGC-3′ and reverse 5′-TCAATTGACCACTCGCACAC-3′. Primer sets for *NOTCH1*, *NOTCH2*, and *ACTB* were published elsewhere (Hubmann et al., 2013). Real-time quantitative RT-PCR (qPCR) for *NOTCH2* was performed with TaqMan^®^-probes (Hs01050717_m1) purchased from Applied Biosystems (Thermo Fisher Scientific, Waltham, MA, United States).

### Gene Silencing by RNA-Interference

siRNA duplexes (siRNAs) for *NOTCH2* (ON-TARGETplus^TM^) and controls (RISC-free Co-siRNA, and siGLO red transfection indicator) were obtained from Dharmacon (Lafayette, CO, United States). Transfection of siRNAs into HCC cell lines was performed by using the lipid reagent siLentFect^TM^ from Bio-Rad Laboratories. The transfection efficiency was determined by FACS and varied from 80 to 90%.

### Cellular Reactive Oxygen Species (ROS) Detection Assay

The redox status was analyzed by a DCFDA (2′,7′-dichlorofluorescin diacetate) containing ROS detection assay using a kit from Abcam (Cambridge, United Kingdom) according to the manufacturer’s instructions. Within the cells, DCFDA is deacetylated and oxidized by ROS into highly fluorescent DCF (2′,7′-dichlorofluorescin) which is measured by flow cytometry.

### Tumor Xenograft Model

Pathogen-free, 4–6 week old, female athymic nude mice (Harlan Winkelmann, Borchen, Germany) were housed under sterile conditions and treated according to the regulations of the local animal ethics committee (BMBWK-66.009/0055-BrGT/2006). Hundred microliter of a tumor cell suspension in PBS containing 9 × 10^6^ 518A2 cells were inoculated subcutaneously into the lower right and left flank of each mouse as described previously (Krepler et al., 2004). When mean tumor volume reached approximately 75 mm^3^ (based on caliper measurements), mice were randomly assigned to treatment groups. One hundred and fifty microliter gliotoxin solution or an equal amount of vehicle control (Vitamin-E derivative) was intraperitoneal injected according to the dosing schedule. The trial was stopped when control mice reached a mean tumor volume of 1 cm^3^.

### Statistics

Statistical significance of differences among treatment groups was calculated by one-way ANOVA and Bonferroni’s test using SPSS software (SPSS Inc., Chicago, IL, United States). Differences in mean tumor volume between two time points within treatment groups were calculated by using the Wilcoxon matched pairs test. *P*-values less than 0.05 were considered to be of statistical significance.

## Results

### Gliotoxin But Not DAPT Inhibited DNA Binding of NOTCH2 and Induced Apoptosis in Cell lines of Human Solid Tumors

**FIGURE 1:**
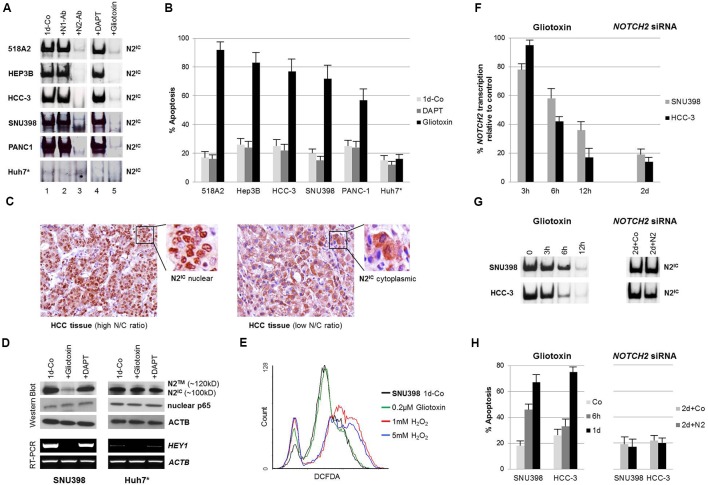
Gliotoxin selectively induces apoptosis in nuclear NOTCH2/CSL active cell lines of solid tumor origin. **(A)** Electrophoretic mobility shift assays (EMSA) and supershift/interference assays conducted with antibodies specific for N1^IC^ (bTAN 20, N1-Ab) and N2^IC^ (C651.6DbHN, N2-Ab) revealed that NOTCH2 is the dominant active NOTCH family member bound on CSL sites in the indicated cell lines (left panel). Cells were incubated with 5 μM DAPT or 0.2 μM gliotoxin for 1 day and the sensitivity of DNA-bound N2^IC^ complexes to the compounds was determined by EMSA (right panel). **(B)** Corresponding FACS analysis showing the effect of 5 μM DAPT and 0.2 μM gliotoxin on apoptosis. **(C)** Immunohistochemistry, showing the nuclear/cytoplasmic localisation of NOTCH2 in HCC tissues in relation to the N/C ratio. **(D)** Western blotting and RT-PCR showing the effect of gliotoxin (0.2 μM) and DAPT (5 μM) on the expression of NOTCH2 and its target gene *HEY1* in nuclear N2^IC^ positive SNU398 and nuclear N2^IC^ negative Huh7 HCC cells after 1 day of incubation. Nuclear NFκB p65, a redox sensitive transcription factor, was not affected by gliotoxin and DAPT. **(E)** 0.2 μM gliotoxin did not induce oxidative stress in SNU398 cells. Cells were treated with 0.2 μM gliotoxin and with two different concentrations of H_2_O_2_ and the ROS concentration was determined by a DCFDA assay via flow cytometry. **(F–H)** Quantitative RT-PCR (qPCR), EMSA, and FACS comparing the time dependent effect of NOTCH2 inhibition by gliotoxin and siRNA on *NOTCH2* mRNA expression, NOTCH2/CSL complexes, and on apoptosis in SNU398 and HCC-3 cells. Data is given as mean from three independent experiments ± standard deviation. ^∗^The nuclear NOTCH2 negative HCC cell line Huh7 cells served as negative control.

The oncogenic properties of NOTCH receptors are tightly linked with their nuclear localization and their transcriptional activity (Jeffries and Capobianco, 2000; Tando et al., 2013; Ressel et al., 2014; Saito et al., 2016). In the nucleus, the intracellular domain of NOTCH (N^IC^) forms a ternary transcription factor complex on CSL responsive genes (Ntziachristos et al., 2014). Therefore, we analyzed nuclear extracts from human cell lines derived from melanoma (518A2), HCC (Hep3B, HCC-3, SNU398, Huh7), and pancreas-CA (PANC-1) for DNA-bound NOTCH/CSL complexes on a CSL consensus site (GTGGGAA) spanning probe by EMSA. We found that NOTCH is highly active in these cell lines with exception of Huh7 (**Figure 1A**, lane 1). Addition of N2-Ab to the EMSA reaction (supershift/interference assay) completely disrupted the formation of the transcription factor complexes (**Figure 1A**, lane 3) whereas N1-Ab had no effect (**Figure 1A**, lane 2). This shows that NOTCH2 is the dominant nuclear active NOTCH receptor in these cell lines. Interestingly, the well differentiated HCC cell line Huh7 did not display any DNA-bound NOTCH complexes and, thus, served as negative control for canonical NOTCH/CSL signaling in our experiments (Hayashi et al., 2015).

In order to analyze the efficiency of GSI in these cell lines, we next tested the sensitivity of DNA-bound NOTCH2 complexes to the widely used and selective GSI DAPT. As shown in **Figure 1A** lane 4, treatment of 518A2, HEP3B, HCC-3, SNU398, and PANC-1 cells with 5 μM DAPT, a concentration which we previously showed that it inhibits NOTCH2 signaling in GSI sensitive CLL cases (Hubmann et al., 2010, 2013), had no effect on the NOTCH2 transcription factor complex after 1 day of incubation.

Based on our recent data which showed that the *Aspergillum* derived secondary metabolite gliotoxin is a potent NOTCH2/CSL transactivation inhibitor in CLL cells (Hubmann et al., 2013), we tested the effect of gliotoxin in these cell lines. As shown in **Figure 1A** lane 5, exposure to gliotoxin at 0.2 μM (=64 ng/ml) for 24 h completely blocked the formation of DNA-bound N2^IC^ complexes in nuclear NOTCH2 positive cell lines.

In terms of cell viability, DAPT had almost no effect while gliotoxin efficiently induced apoptosis. Interestingly, the pro-apoptotic effect of gliotoxin was restricted to nuclear NOTCH2 positive cells while the well differentiated nuclear NOTCH2 negative HCC cell line Huh7 was found to be resistant to gliotoxin treatment (**Figure 1B**).

Since NOTCH2 is considered as a tumor suppressor gene in certain breast cancer cell lines (O’Neill et al., 2007; Kim et al., 2016), we additionally tested HCC38 and MDA-MB-468 breast cancer cells for their NOTCH activity and sensitivity to gliotoxin. HCC38 cells were found to be positive for NOTCH2/CSL complexes whereas no remarkable NOTCH activity could be detected in MDA-MB-468 cells (Supplementary Figure 1A). In HCC38 cells, gliotoxin inhibited NOTCH2/CSL complexes (supplementary Figure 1A) and induced apoptosis in a dose dependent manner (Supplementary Figure 1B) whereas MDA-MB-468 cells were resistant to gliotoxin treatment (Supplementary Figure 1B).

In order to substantiate the relation between the differentiation grade and the nuclear localization of NOTCH2, we performed immunohistochemical analysis of human HCC tissue sections obtained from patients with less differentiated and more differentiated tumors. In accordance with published data (Hayashi et al., 2015), staining primary human HCC tissues with anti-NOTCH2 antibodies showed a prominent nuclear localization of NOTCH2 in less differentiated HCC tissues with a more immature cellular morphology as indicated by a high nuclear to cytosol (N/C) ratio. In contrast, a predominant cytoplasmic localization of NOTCH2 was found in more differentiated HCC tissues with low H/C ratio, underlining the clinical relevance of our cell line data (**Figure 1C**). A global overview about the expression and localization of NOTCH2 in cancer tissue arrays is presented in the human protein atlas^1^ (Uhlen et al., 2015).

### Gliotoxin Selectively Targets NOTCH2 Expression in Nuclear N2^IC^ Active HCC Cells and Induces Apoptosis Independent of the Redox Status

We next tested the effect of gliotoxin on total NOTCH2 protein expression in nuclear NOTCH2 positive (SNU398) and in nuclear NOTCH2 negative (Huh7) HCC cells by western blotting. The wild type NOTCH2 receptor is anchored as cleaved heterodimer on the cell surface. This consists of a 180-kD NOTCH2 extracellular domain (N2^EC^) and a 120-kD NOTCH2 transmembrane form (N2^TM^). After ligand binding, the 100-kD NOTCH2 intracellular domain (N2^IC^) is released from the N2^TM^ form by γ-secretase cleavage. As shown in **Figure 1D**, both cell lines express mainly N2^IC^ (100-kD) whereas N2^TM^ (120-kD) was only weakly detectable in Huh7 cells (**Figure 1D**). Gliotoxin efficiently inhibited the expression of NOTCH2 in SNU398 cells but not in Huh7 cells suggesting that gliotoxin specifically targets NOTCH2 expression in nuclear N2^IC^ active SNU398 cells. However, further studies on the subcellular localization of NOTCH2 and dynamics of NOTCH2 migration upon exposure to gliotoxin would be of a major interest for getting deeper insight on the mechanism of action of gliotoxin in terms of NOTCH regulation.

The nuclear NOTCH2 activity in SNU398 cells was reflected by the transcription of its target gene *HEY1* (**Figure 1D**). Gliotoxin but not DAPT inhibited *HEY1* transcription in SNU398 cells. This result supports the hypothesis, that deregulation of NOTCH2 signaling in certain cell lines is probably caused by the expression of truncated, ligand independent N2^IC^ forms which do not require γ-secretase for processing and function.

Since gliotoxin may have a wide range of downstream targets (Scharf et al., 2016) and may exert its apoptotic effect via reactive oxygen species (ROS) production and via inhibition of the redox sensitive transcription factor NFκB (Gardiner et al., 2005), we evaluated the effect of gliotoxin on the nuclear expression of the NFκB subunit p65 (RelA) (Gloire and Piette, 2009). As shown in **Figure 1D**, gliotoxin had no influence on the expression of p65 in nuclear extracts of both cell lines. Furthermore, gliotoxin did not influence the redox status in gliotoxin sensitive SNU398 cells as determined by a cellular ROS detection assay (**Figure 1E**). In summary, these results clearly show that the induction of apoptosis by gliotoxin, at least in this cell line, is associated with the transcriptional NOTCH2/CSL activity, is independent of the GSI sensitivity of N2^IC^, and is not associated with effects on the redox status of the treated cells.

We next compared the effect of *NOTCH2* inhibition by gliotoxin and siRNA in the HCC cell lines SNU398 and HCC-3. Gliotoxin remarkably inhibited *NOTCH2* mRNA expression (**Figure 1F**, left panel), the formation of NOTCH2/CSL complexes (**Figure 1G**, left panel), and induced apoptosis (**Figure 1H**, left panel) within 12 h of incubation. In contrast, *NOTCH2* siRNA downregulated *NOTCH2* mRNA expression (**Figure 1F**, right panel) but had no influence neither on NOTCH2/CSL complexes (**Figure 1G**, right panel) nor on cell viability (**Figure 1H**, right panel) within 2 days of incubation. This indicates that gliotoxin may target NOTCH2 signaling at the transcription factor level which in turn disrupts a positive feedback loop of *NOTCH2* mRNA expression (Artavanis-Tsakonas et al., 1999). In contrast, downregulation of *NOTCH2* mRNA alone seems to be insufficient to target NOTCH2 signaling. This might be explained by the stability of the NOTCH2/CSL complex on DNA. Together, these results confirmed that the induction of apoptosis by gliotoxin is tightly linked with its inhibitory effect on the formation of the NOTCH2/CSL complex on DNA (Hubmann et al., 2013).

### Gliotoxin Is Effective in a Human Melanoma Xenograft Mouse Model

Since many *in vivo* investigations and clinical trials with GSI have been already reported (Espinoza and Miele, 2013; Andersson and Lendahl, 2014) or are still ongoing^2^, we focused the *in vivo* investigation on gliotoxin treatment using our established 518A2 melanoma xenograft mouse model (Krepler et al., 2004).

**FIGURE 2:**
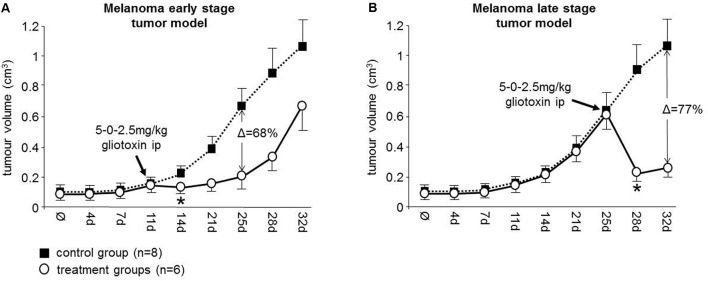
Gliotoxin efficiently targets melanoma tumors in athymic nude mice. A single day dosing schedule (5-0-2.5 mg/kg) of gliotoxin was intraperitoneally applied to 518A2 xenotransplanted mice **(A)** on day 11 (group A, early stage tumor model) and **(B)** on day 25 (group B, late stage tumor model) as indicated. The effect of gliotoxin on the tumor volume in the treatment groups (indicated as white circles) relative to the control group (indicated as black squares) was determined by serial caliper measurements. Data is given as mean ± 95% confidence interval. Δ values indicate the maximum percentage of tumor mass reduction relative to controls mediated by gliotoxin in these two groups. ^∗^Denotes the beginning of statistical significance.

We found that intraperitoneal (ip) application of gliotoxin on a single day (5 mg/kg in the morning and 2.5 mg/kg in the evening) was well tolerated and the mice did not show any study limiting side effects. Therefore, we applied this gliotoxin dosing schedule to the treatment groups on day 11 (early stage tumor model; group A, *n* = 6) and on day 25 (late stage tumor model; group B, *n* = 6), respectively. We then determined the effect of gliotoxin on the tumor mass in treated animals compared to the control group (*n* = 8) by serial caliper measurements (**Figure 2**).

In the early stage tumor model, application of gliotoxin on day 11 revealed a significant decrease of the mean tumor volume on day 14 (control group versus treatment group A: 115 mm^3^ vs. 83 mm^3^; *P* = 0.008) with the greatest tumor mass reduction after 2 weeks (68%) (**Figure 2A**). This effect was even more pronounced in the late stage tumor model, where the same dosing schedule was applied on day 25 (**Figure 2B**). This caused a significant decrease of the mean tumor volume on day 28 (control group versus treatment group B: 576 mm^3^ vs. 218 mm^3^, *P* = 0.005) with the greatest tumor mass reduction after 1 week (77%) (**Figure 2B**). Therefore, we conclude that gliotoxin is highly active in this melanoma xenograft mouse model and, thus, is therapeutically relevant for NOTCH2 associated malignancies.

## Discussion

Although human cancers evolve by the progressive accumulation of driver mutations in genes with diverse functions, they may be highly dependent on a singular tumor initiating oncogene (oncogene addiction) (Weinstein and Joe, 2008). Such oncogenes have the potential to serve as “Achilles heel” for a targeted therapy. Members of the NOTCH gene family are deregulated in a wide variety of human neoplasias making NOTCH a promising candidate for therapeutic interventions. The oncogenic characteristics of NOTCH receptors are tightly linked with their nuclear localization and their transcriptional activity (Jeffries and Capobianco, 2000; Tando et al., 2013; Ressel et al., 2014; Saito et al., 2016). Due to the lack of reliable, cell type independent NOTCH target genes (Ntziachristos et al., 2014), we used EMSA as a surrogate model for the detection of NOTCH/CSL transcription factor complexes and found that human cell lines derived from melanoma (518A2), HCC (Hep3B, HCC-3, SNU398), and pancreas-CA (PANC-1) express high amounts of nuclear NOTCH2.

One strategy to target aberrant NOTCH signaling is the use of GSI which are currently tested in many clinical trials (Espinoza and Miele, 2013; Andersson and Lendahl, 2014). However, the outcomes of these studies are often disappointing which might be explained by the expression of truncated, ligand independent NOTCH forms which do not require γ-secretase for processing and function (Lauring and Overbaugh, 2000; Das et al., 2004; Ntziachristos et al., 2014), and/or by GSI mediated effects on the tumor microenvironment as has been recently shown in an immunocompetent C57BL/6 mouse model (Dai et al., 2017). Moreover, the nuclear NOTCH2/CSL complexes might be relatively stable as indicated by siRNA mediated inhibition of *NOTCH2* mRNA expression which had no influence on the amount of NOTCH2/CSL complexes in HCC cell lines *in vitro* as shown in this work.

In line with this hypothesis, we show that DNA-bound N2^IC^ complexes as well as total N2^IC^ expression are resistant to GSI treatment in the analyzed cell lines. Therefore we tested alternatively the therapeutic potential of gliotoxin which is a potent inhibitor of canonical NOTCH2/CSL signaling and which also induced apoptosis in CLL cells under microenvironment conditions in co-culture with primary bone marrow stromal cells (Shehata et al., 2010; Hubmann et al., 2013). We confirmed that gliotoxin completely blocked DNA-bound N2^IC^ complexes in all tested cell lines (Hubmann et al., 2013). The specificity of gliotoxin was demonstrated in the well differentiated HCC cell line Huh7 (Hayashi et al., 2015) and in the breast cancer derived cell line MDA-MB-468, which did not display a detectable nuclear NOTCH/CSL activity and which were found to be resistant to gliotoxin treatment *in vitro*. Moreover, gliotoxin did not influence the redox status and the nuclear expression of the redox sensitive NFκB p65 (RelA) subunit in SNU398 cells (Gardiner et al., 2005; Gloire and Piette, 2009). Thus, the data may confirm that gliotoxin exerts its apoptotic effect primarily via targeting canonical NOTCH2/CSL signaling. However, more work need to be done for a better understanding of the effect of gliotoxin on the complex and interconnected signal transduction pathways in cancer cells. Moreover, a large scale screening process on a wide range of tumors and tumor subtypes should identify nuclear NOTCH2/CSL positive entities that might respond to gliotoxin treatment.

Since the therapeutic options for melanoma, pancreas-CA, and HCC patients are limited, we evaluated the clinical relevance of gliotoxin *in vivo*. Because DAPT had no effect on NOTCH2/CSL complexes in the cell lines tested, we focused on evaluating the effect of gliotoxin *in vivo*. However, a comparative *in vivo* study using gliotoxin and GSI in parallel needs to be taken into consideration. A single day dosing schedule of gliotoxin led to a significant tumor mass reduction in an early stage (68%) as well as in a late stage (77%) melanoma xenograft mouse model. In accordance with other animal studies (Wright et al., 2001; Vigushin et al., 2004; Nejak-Bowen et al., 2013), the applied gliotoxin dose was well tolerated and far lower than its reported toxic doses (Richard, 1990). Based on the body surface area (BSA) indices, the human equivalent dose (HED) of 5 mg/kg gliotoxin used in this study would be as low as 0.405 mg/kg (Reagan-Shaw et al., 2008). In addition, the effective concentration of gliotoxin *in vitro* (0.2 μM ≙ 64 ng/ml) is still up to 10 times lower than its serum concentrations detected in patients suffering from aspergillosis (Lewis et al., 2005) pointing to the physiological relevance of our data. One has to consider, however, that xenotransplanted cell lines in immunocompromised mice will not fully reflect the situation expected in human tumors or precisely predict the outcome in patients and therefore, careful approaches should be taken to justify clinical evaluation in human.

Taking together, this work shows that targeting canonical NOTCH2/CSL signaling by gliotoxin is associated with the induction of apoptosis in cell lines derived from melanoma, HCC, and pancreas-CA whereas the GSI DAPT is ineffective. The potential clinical relevance of this finding is demonstrated in a melanoma xenograft mouse model, showing that gliotoxin significantly reduced the tumor volume in early as well as in late stage tumors. Although the available preclinical data presented in this work are encouraging, more evidence needs to be directly demonstrated on human tumors. However, this proof of concept serves as a perspective and may justify further explorations of the therapeutic potential of gliotoxin in NOTCH2 associated human neoplasias.

## Author Contributions

RH, MS, and WS designed the study, performed the *in vitro* and animal experiments, analyzed and interpreted the data, wrote the manuscript and approved the final version. SS, MA, MH, MR, and DD contributed substantially to the experimental work, data analysis and interpretation, revision and approval of the manuscript. PV, CZ, and UJ contributed to data analysis and interpretation, critically reviewed and approved the manuscript.

## Conflict of Interest Statement

RH, WS, and MS own a patent on the usage of gliotoxin as a tool for therapy for NOTCH2 associated malignancies (US Patent No. 7,981,878). The other authors declare that the research was conducted in the absence of any commercial or financial relationships that could be construed as a potential conflict of interest.

## Footnotes

1 http://www.proteinatlas.org

2 https://clinicaltrials.gov/
