# Supplementary figures and images for: Gliotoxin Targets Nuclear NOTCH2 in Human Solid Tumor Derived Cell Lines In Vitro and Inhibits Melanoma Growth in Xenograft Mouse Model
Source: Front Pharmacol. 2017 Jul 7;8:319. doi: 10.3389/fphar.2017.00319 (PMC5500618; doi:10.3389/fphar.2017.00319)

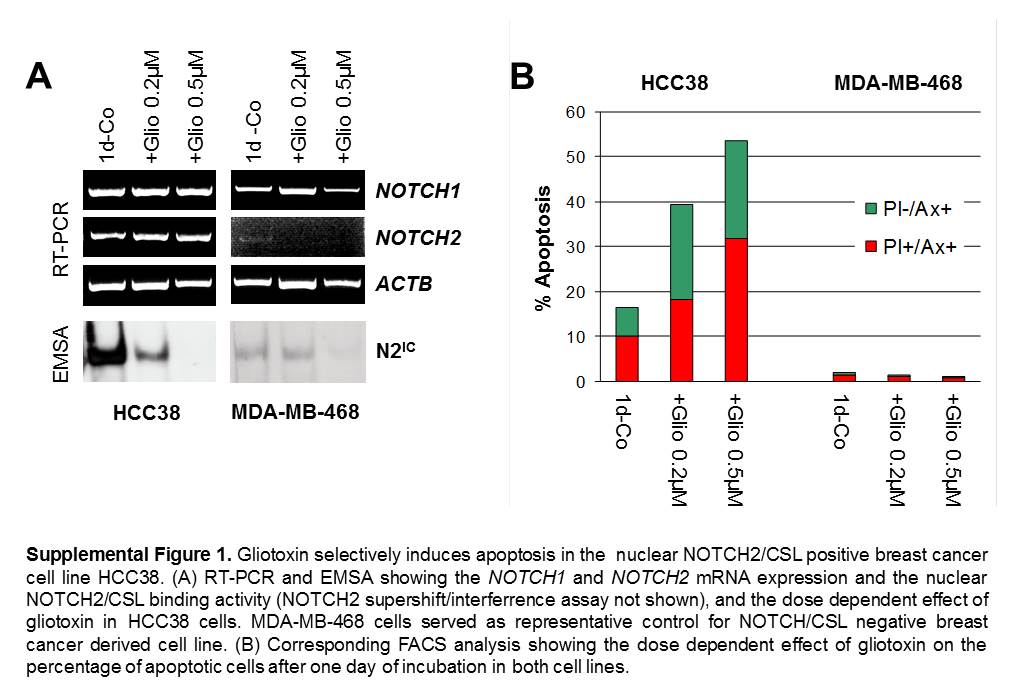

Supplement: Supplementary file 1 [file Image_1.JPEG]
